# A translational colorectal cancer organoid biobank mirrors patients’ tumor histology, molecular profiles, and treatment responses

**DOI:** 10.1186/s13046-026-03666-x

**Published:** 2026-02-25

**Authors:** Moritz Jesinghaus, Miguel Gomes Silva, Antonio Enrico Zaurito, Valentina Brunner, Frederic Saab, Anantharamanan Rajamani, Niklas de Andrade Krätzig, Nicholas Bodenstein, Rémi Guillemant, Junika Pohl, Maxime Schmitt, Rupert Öllinger, Federico Fusco, Julius Shakhtour, Peter Klare, Klaus-Peter Janssen, Sebastian Foersch, Katja Steiger, Roland Rad, Nicole Pfarr, Dieter Saur, Markus Tschurtschenthaler

**Affiliations:** 1 Institute of Experimental Cancer Therapy, TUM University Hospital, School of Medicine and Health, Technical University of Munich, Munich, 81675 Germany; 2 Center for Translational Cancer Research (TranslaTUM), School of Medicine and Health, Technical University of Munich, Munich, 81675 Germany; 3 Institute of Pathology, School of Medicine and Health, Technical University of Munich, Munich, 81675 Germany; 4 Institute of Pathology, University Hospital Marburg, Marburg, 35043 Germany; 5 Institute of Molecular Oncology and Functional Genomics, School of Medicine and Health, Technical University of Munich, Munich, 81675 Germany; 6 Division of Translational Cancer Research German Cancer Research Center (DKFZ) and German Cancer Consortium (DKTK), Heidelberg, 69120 Germany; 7 Department of Medicine II, School of Medicine and Health, TUM University Hospital, Technical University of Munich, Munich, 81675 Germany; 8 Department of Surgery, School of Medicine and Health, Technical University of Munich, Munich, 81675 Germany; 9 Institute of Pathology, University Medical Center, Johannes Gutenberg University Mainz, Mainz, 55101 Germany; 10 German Cancer Consortium (DKTK), Partner Site Munich, Munich, Germany

**Keywords:** Colorectal cancer, Patient-derived organoids (PDOs), Histological subtypes, Tumor heterogeneity, PDOX models

## Abstract

**Background:**

Colorectal cancer (CRC) exhibits pronounced inter- and intratumoral heterogeneity, emphasizing the need for preclinical models that accurately capture its molecular and histological diversity. Patient-derived organoids (PDOs) represent valuable ex vivo systems to model CRC, yet whether they can preserve subtype-specific features and maintain fidelity upon in vivo transplantation remains unclear.

**Methods:**

We established a biobank of PDOs from both treatment-naïve and neoadjuvant-treated CRC patients, encompassing the major histological subtypes – micropapillary, medullary, serrated, mucinous, and adenocarcinoma not otherwise specified (NOS). PDOs were comprehensively characterized by histomorphological, genomic, and transcriptomic analyses. To assess in vivo fidelity, PDOs were orthotopically transplanted into immunodeficient mice to generate patient-derived organoid xenografts (PDOXs). PDOX-derived tumors and organoids were analyzed to evaluate the preservation of histological and molecular traits, as well as therapy responses.

**Results:**

PDOs displayed distinct, subtype-specific morphologies and growth patterns that closely paralleled their respective patient tumor histologies. Orthotopic PDOXs recapitulated the histological architecture, gene expression profiles, and signaling pathway activation of the original tumors. PDOX-derived organoids retained these subtype-specific morphologies, molecular features, and exhibited similar responses to FOLFOX treatment as their corresponding PDOs, confirming both molecular and functional stability of the organoid-xenograft cycle.

**Conclusion:**

This study establishes orthotopic transplantation of CRC PDOs as a robust and predictive preclinical model that captures the full spectrum of CRC heterogeneity. The model preserves histological and molecular subtype fidelity across in vitro and in vivo contexts and enables functional assessment of therapy response. By bridging patient-derived tumor biology with translational modeling, this platform provides a valuable resource for dissecting CRC pathogenesis and advancing patient-tailored precision oncology.

**Graphical Abstract:**

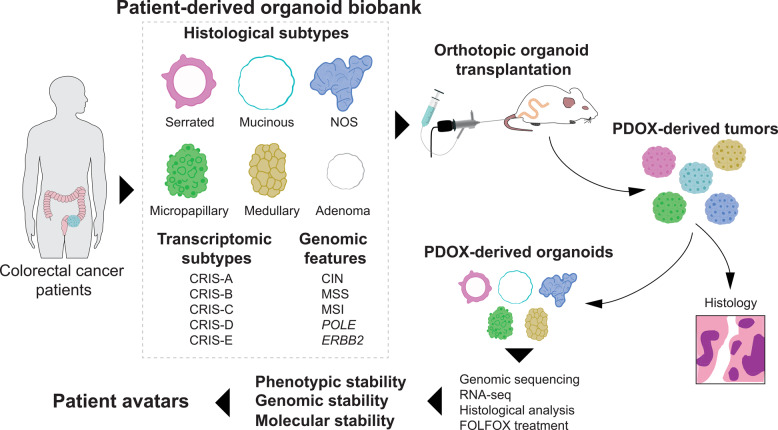

**Supplementary Information:**

The online version contains supplementary material available at 10.1186/s13046-026-03666-x.

## Introduction

Colorectal cancer (CRC) is one of the most prevalent cancers worldwide and is associated with high morbidity and mortality [1, 2]. The pronounced inter- and intratumoral morphomolecular heterogeneity of CRC gives rise to a distinct histological and molecular profile in every patient, complicating accurate predictions of disease course and treatment response [3].

On the one hand, this inter- and intratumoral heterogeneity manifests histologically through a broad spectrum of morphological patterns, ranging from differences in gland formation to variations in tumor cell dissociation and to the general histological architecture. The current 5th edition of the WHO Classification, considered the international gold standard for histopathological evaluation, recommends the assessment of three ‘essential and desirable’ morphological criteria on hematoxylin and eosin (H&E)-stained slides for routine diagnostic reporting [4, 5]. Previous studies have confirmed the profound prognostic significance of these factors [5–7]. These criteria include tumor budding, defined as invasive clusters of fewer than five cancer cells, which serves as a marker of the tumor’s dissociative growth potential and is categorized into three grades (Bd1, Bd2, Bd3) according to international consensus guidelines [7, 8]. Tumor grade, which reflects the extent of glandular formation, is another key parameter [8]. Finally, the classification also considers the general histopathological subtype, which captures the tumor’s overall architectural pattern. The most common subtype is adenocarcinoma not otherwise specified (NOS), followed by mucinous, micropapillary, and serrated adenocarcinoma [6].

On the other hand, this heterogeneity is equally evident at the molecular level. Genetically, at least three major evolutionary pathways have been described: chromosomal instability (CIN), microsatellite instability (MSI), and the ultramutator phenotype [1, 9]. In addition, various transcriptional studies have identified distinct gene expression signatures. Molecular subtyping efforts, such as the Consensus Molecular Subtypes (CMS) and the CRC Intrinsic Subtypes (CRIS) classifications, aim to integrate these molecular characteristics into biologically meaningful categories [10, 11]. However, despite their potential, these classification systems have not yet been widely adopted in clinical routine [1, 9, 10].

This pronounced morphological and molecular heterogeneity is not only challenging for accurate diagnosis and prognosis of CRC, but also limits the utility of the most commonly used preclinical models, such as patient-derived 2D cell lines and patient-derived xenografts (PDXs), which often fail to capture the functional complexity of individual tumors. The establishment of 2D cell lines is generally inefficient, and adherent cells rarely preserve the original tumor architecture [12, 13]. PDX models provide a more physiological alternative by transplanting human tumor cells into mice [14–16], but they frequently undergo genetic drift during in vivo propagation, potentially altering tumor behavior and reducing predictive accuracy [14, 17, 18]. Therefore, reliable systems are urgently needed to improve the prediction of therapeutic response.

In recent years, patient-derived organoids (PDOs) have emerged as a powerful preclinical model that overcomes several limitations of traditional in vitro and in vivo systems. PDOs can be derived from both normal mucosa and tumors, reliably recapitulating key molecular features of the original patient tissue [19–23]. They have significantly advanced our understanding of disease phenotypes [24, 25] and epithelial interactions within the tumor microenvironment [26–28]. Moreover, PDOs provide a valuable platform for drug screening and therapy development, demonstrating predictive value for both targeted and conventional therapies, including chemotherapy and radiotherapy [22, 29–37]. Their potential in guiding personalized treatment strategies has led to their integration into clinical trials [38], underscoring their translational relevance in precision oncology.

However, PDOs are limited in their ability to predict complex therapy responses and disease progression due to their purely in vitro nature. Moreover, little is known about how these models evolve within a living environment that resembles their tissue of origin. These limitations underscore the need for systems that combine the physiological relevance of PDXs with the scalability and molecular fidelity of PDOs. To address this, orthotopic mouse transplantation models – where PDOs are implanted and challenged within a more native microenvironment – have emerged as a promising strategy to bridge this gap, providing a physiological context that more accurately reflects tumor behavior in vivo [24, 39–41].

In the present study, we established an orthotopic human organoid transplantation model incorporating PDOs derived from a comprehensive CRC organoid biobank representing the most common histological subtypes of CRC. Organoids were endoscopically transplanted into the rectum of NOD scid gamma (NSG) mice, leading to the development of transplantation-derived carcinomas, including metastases. We performed an in-depth histological and molecular comparison between the original patient tumors, corresponding PDOs, and the resulting implantation-derived carcinomas. Furthermore, second-generation organoids were generated from the in vivo (xenograft) tumors. Notably, key morphological features remained remarkably stable after transplantation and were accompanied by the preservation of major molecular alterations. Exploratory drug screening experiments revealed comparable treatment responses across all PDO-derived systems and the corresponding patient tissue. To our knowledge, this is the first study to systematically assess the phenotypic stability of different CRC subtypes at histological, genomic, and molecular levels – both in vitro and after in vivo passaging.

## Methods

### Animal studies

Immunocompromised NSG (NOD.Cg-*Prkdc*^scid^ *Il2rg*^tm1Wjl^/SzJ, Stock No: 005557) mice were purchased from Charles River Laboratories (Research Models and Services, Germany GmbH, Sulzfeld, Baden-Württemberg, Germany) and housed, bred and maintained in specific pathogen-free (SPF) facilities (ZPF, TUM University Hospital, Technical University of Munich, Germany). All animal studies were conducted in compliance with European guidelines for the care and use of laboratory animals and were institutionally approved by the Institutional Animal Care and Use Committees (IACUC) of Technical University of Munich and by the District Government of Upper Bavaria.

### Patient samples

All tumors used in this study were obtained post-surgical resection using sterile instruments. Upon excision, they were immediately transported to the laboratory in ice-cold PBS for tumor cell isolation and organoid cultures. All experiments were approved by the ethics committee vote 458/17 S (Amendment: 256/18 S) of the TUM University Hospital of the Technical University of Munich.

### Orthotopic organoid transplantation

Mice were anesthetized using midazolam (5.0 mg/kg), medetomidine (0.5 mg/kg), and fentanyl (0.05 mg/kg). Anesthetized mice were placed ventral side down on a heating pad and the distal colon was gently rinsed with PBS using a 1 mL syringe and a straight gavage needle (Fine Science Tools; 20-gauge, 1.25 mm tip diameter, 30 mm length). Orthotopic transplantations of human tumor organoids into recipient mice were performed as previously described [41, 42]. Briefly, organoids were mechanically dissociated into 5–10 cell clusters and resuspended in PBS containing 10% Matrigel (Corning), 1x B27, 1x N2, 100 U/mL of Pen/Strep (all from Gibco, Thermo Fisher Scientific) and 10µM Y-27632 (STEMCELL Technologies). For every injection (2–3 per mouse) ~ 50 dissociated organoids in a volume of 100 µl were prepared. All organoids used for transplantation in this study were passaged in vitro fewer than 12 times.

Mouse colonoscopy was performed using a rigid endoscope from Karl STORZ (1.9 mm in diameter) with a linear Hopkins lens optics (ColoView System). An external air pump (EHEIM) was used to allow more precise regulation of the airflow than the endoscope’s integrated airflow system. For injections of organoids into the submucosa of the colon a flexible fine needle (Hamilton; 33-gauge, custom length of 16 inches, custom point style of 4 at 45°) was used. Injections that were correctly applied into the submucosa led to the formation of a bubble that closes the intestinal lumen. Bubble size and stability were graded according to an internal evaluation scheme. After 2–3 injections, the mice were antagonized with atipamezole (2.5 mg/kg), flumazenil (0.5 mg/kg), and naloxone (1.2 mg/kg) and maintained on a heating pad until activity level increased.

All animals with signs of sickness were sacrificed in compliance with the European guidelines for the care and use of laboratory animals. For necropsy of tumor-bearing mice, the large intestine and colon was macroscopically checked for the presence of primary tumors and metastases at the main metastatic routes (liver, lung, lymph nodes).

### Tumor cell isolation and organoid cultures

Fresh tumor tissue samples were processed as previously described [20] with some modifications. Tumor material pieces were minced with scalpels into smaller pieces, digested in a solution containing 5 mL of Dispase II solution (ready to use, Millipore), 100 U/mL Collagenase IV (Sigma-Aldrich) and 20 U/mL of DNaseI solution (Sigma-Aldrich) and incubated at 37 °C for 60 min. Tissue suspension is then filtered using a 70 μm cell strainer (EASYstrainer, Greiner bio-One) and a total of 100–150 cell clumps are resuspended in Matrigel plated in a well of a 24-well plate supplemented with a medium containing Advanced DMEM/F12, 10 mM HEPES, 2 mM Glutamax, 1x B27 (all from Gibco, Thermo Fisher Scientific) 1.25 mM N-Acetylcysteine (Sigma-Aldrich), 50 ng/mL human EGF, 100 ng/mL human Noggin, 1 µg/mL human R-Spondin 1, 100 ng/ml Wnt3A (all from Peprotech), 500 nM A83-01, 7.5 µM SB202190, 10 mM Nicotinamide (all from Sigma-Aldrich), 100 µg/mL Normocin (InvivoGen), 1x Antibiotic-Antimycotic (Gibco, Thermo Fisher Scientific), 10 µM Y-27632 (STEMCELL Technologies). Passaging of organoid cultures was done by incubating the culture with Cell Recovery Solution (Corning) for 30 min on ice to dissolve the Matrigel and retrieve the cellular part of the solution. Cells were the chemically digested by incubation with TypLE Express Enzyme (Gibco, Thermo Fisher Scientific) diluted 1:1 in PBS and incubated at 37 °C for 3 min. Dissociated organoids were subsequently seeded in Matrigel and cultured in the previously described medium. Organoids were frozen and biobanked in Recovery Cell Culture Freezing Medium (Gibco, Thermo Fisher Scientific). Organoid cultures were routinely tested for mycoplasma by PCR analysis.

### Genomic DNA and RNA isolation

The content of 3 dense 24-wells with organoids was used for the isolation of either genomic DNA (gDNA) using the GenElute Mammalian Genomic DNA Miniprep Kit (Sigma-Aldrich) or RNA using the RNeasy Mini kit (Qiagen) according to manufacturer’s instructions. Briefly, organoids were grown up to 7 days in Matrigel in a 24-well plate. On the day of cell harvest, cultures were incubated with Cell Recovery Solution for separation of Matrigel and cellular part. Cell pellets were snap frozen in liquid N_2_ and stored at −80 °C for downstream applications. gDNA and RNA concentrations were determined using a Qubit fluorometer (Thermo Fisher Scientific).

### Fixation and paraffin-embedding of organoids

Organoids cultured in Matrigel on glass coverslips (13 mm diameter, Carl Roth) were fixed in formalin for 45 min at room temperature. After fixation, the Matrigel dome containing the organoids was carefully lifted from the culture plate using a bent needle and transferred into Bionet embedding cassettes (Engelbrecht, Cat. No. 17995). The cassettes were then stored in PBS until dehydration and embedding in paraffin.

### Histopathological assessment

Formalin-fixed paraffin embedded (FFPE) tissues, tissue microarrays (TMAs, consisting of 1 mm triplicate tissue cores) and organoids were sectioned at 2 μm slices with a microtome. For seven matched cases of human carcinomas and their corresponding patient-derived orthotopic xenograft (PDOX) tumors. TMAs were constructed as described previously [43–45], comprising three to six tumor cores per lesion. Tissue slices were subjected to deparaffinization and rehydration prior to histopathological analysis. For hematoxylin and eosin (H&E) staining, experiments were subsequently conducted following standard protocols. Histopathologic assessment of the patient tumors as well as the PDOX-derived carcinomas was performed by an experienced gastrointestinal pathologist (M.J.). All human and PDOX-derived carcinomas were examined to assess the distribution and prevalence of the morphology-based “*essential and desirable criteria*” outlined in the 2019 WHO classification of digestive system tumors, specifically tumor budding, tumor grade, and histological subtype [4]. Tumor budding was defined as the presence of single cells or clusters of fewer than five cells at the invasive front. It was evaluated on H&E-stained slides using a three-tiered scoring system: Bd1 (low budding activity: 0–4 buds), Bd2 (intermediate budding activity: 5–9 buds), and Bd3 (high budding activity: >10 buds) per 0.785 mm^2^ at 20x magnification, following international consensus guidelines [8]. Tumor grade was determined based on gland formation, with tumors categorized as low-grade (≥ 50% gland formation) or high-grade (< 50% gland formation) [4]. The presence of metastatic disease was additionally assessed in mouse liver, lung, pancreas and spleen.

For immunohistochemical staining (IHC), rehydrated tissue slides were pre-treated using heat mediated antigen retrieval with sodium citrate buffer (pH = 6) or enzyme. Primary antibodies included: CDX2 (Abcam), Claudin-1 (Abcam), Desmoplakin (Cell Signaling), E-Cadherin (Leica), CK20 (Progen) and p53 (Agilent Dako). Slides were then incubated at 4 °C for 15 min at room temperature. Antibody detection was performed on the Leica Bond RXm (Leica Microsystems) platform using the BOND Polymer Refine Detection Kit (Leica Microsystems). Stromal content was evaluated using Masson-Goldner trichrome staining. IHC stainings of tumor tissues and organoids were performed at the Comparative Experimental Pathology (CEP) of the TUM University Hospital and at the Institute of Pathology of the University Hospital Marburg. IHC stainings were quantified using the immunoreactivity score (IRS), calculated as the product of staining intensity (0–3) and the proportion of positively stained tumor cells (0–4), yielding a total score ranging from 0 to 12. For lineage markers, CDX2 and CK20 expression were classified as positive or negative according to the percentage of stained tumor cells. p53 expression was categorized as wild-type (WT) or aberrant, with aberrant patterns defined by nuclear overexpression, complete loss of staining, or cytoplasmic mislocalization, consistent with established surrogate markers of *TP53* mutation status [46].

### RNA-sequencing (RNA-seq)

Library preparation for bulk-sequencing of poly(A)-RNA (3-prime RNA) was done as described previously [47]. Briefly, barcoded cDNA of each sample was generated with a Maxima RT polymerase (Thermo Fisher Scientific) using oligo-dT primer containing barcodes, unique molecular identifiers (UMIs) and an adaptor. Ends of the cDNAs were extended by a template switch oligo (TSO) and full-length cDNA was amplified with primers binding to the TSO-site and the adaptor. NEBNext Ultra II FS kit was used to fragment cDNA. After end-repair and A-tailing a TruSeq adapter was ligated, and 3’-end-fragments were finally amplified using primers with Illumina P5 and P7 overhangs. In comparison to Parekh et al. [47] the P5 and P7 sites were exchanged to allow sequencing of the cDNA in read1 and barcodes and UMIs in read2 to achieve a better cluster recognition. The library was sequenced on a NextSeq 550 (Illumina) with 63 cycles for the cDNA in read1 and 16 cycles for the barcodes and UMIs in read2.

### Low-coverage whole genome sequencing (lcWGS)

Low coverage whole genome sequencing (lcWGS) was performed using 200 ng of gDNA from PDOs. Libraries were prepared using the TruSeq DNA Nanokit (Illumina) following the manufacturer’s instructions. Resulting libraries were analyzed on a 2100 Bioanalyzer instrument (Agilent Technologies) and sequenced on a NextSeq 550 (Illumina) or NovaSeq 6000 (Illumina) system.

### Panel sequencing

The identification of mutations in both human FFPE tissue and organoid lines was done by performing targeted amplicon sequencing based on a multiplex PCR-based Ion Torrent AmpliSeq technology (Thermo Fisher Scientific) approach. A custom-designed CRC version 2 panel (CRCv2 panel, Thermo Fisher Scientific) consisting of 2 primer pools for amplification of 379 amplicons spanning covering hot spot exonic regions of 25 genes known to be related to CRC was used (Table 1).

**Table 1:** Custom-designed CRCv2 panel for targeted sequencing of CRC organoids and FFPE tumor samples. The panel covers 379 amplicons spanning hotspot exonic regions (numbers in brackets) of 25 genes implicated in CRC

***AKT1*** (3,4,5,11)	***ERBB2*** (11,12,13,17,19-21)	***MSH6*** (3,4,5,8,9)	***PMS2*** (8,9,10,11)	***SMAD4*** (2-6,8-12)
***APC*** (2-16)	***KRAS*** (2,3,4)	***MYC*** (2,3)	***POLE*** (9-14,26,33,34,36)	***TGFBR2*** (3-7)
***BRAF*** (8,11,14,15,16)	***MED12*** (2)	***NRAS*** (2,3,4)	***PTEN*** (2-10)	***RB1*** (2,3,6,13,16,17,18,20,21,22, 23)
***CTNNB1*** (3,5,6,7,8,15)	***MLH1*** (2,3,4,8,10,12,13,15)	***PIK3CA*** (2,3,5,7-10,12,14,19-21)	***RB1*** (2-27)	***RET*** (10,11,13,15,16)
***EGFR*** (2,3,8,18,19,20,21)	***MSH2*** (1,7,11,13,15)	***PIK3R1*** (2,9,10,11,13,14)	***SMAD2*** (4,5,8,11)	***TP53*** (2,4-10)

Library preparation was performed using the multiplex PCR-based Ion Torrent AmpliSeqTM technology (Thermo Fisher Scientific) applying the custom designed CRC version 2 panel and the Ion AmpliSeq Library Kit v2.0 as described before [48]. Briefly, two pools of primers were generated for the library preparation step. Five nanograms of genomic DNA was used per each pool and mixed with the AmpliSeq HiFi Master Mix (Life Technologies). Individual libraries were diluted to a final concentration of 25 pM and 6 of these libraries were pooled and processed using the Ion S5 510 & 520 & 530 Chef kit on an Ion Chef instrument (both: Thermo Fisher Scientific) where the libraries were processed for sequencing. Sequencing was performed on an Ion S5XL instrument using the Ion S5 Sequencing chemistry and loaded onto a 520 chip.

### FOLFOX treatment

5-Fluorouracil (5-FU, CAS: 51-21−8) and Oxaliplatin (CAS: 61825-94−3; both from Selleckchem) were applied at a 25:1 ratio, as reported previously [35]. Leucovorin was not included, as its primary role in vivo is to modulate drug metabolism in the liver and enhance 5-FU activation – systemic factors that are absent in cell culture. Organoids were seeded in 96-well plates and treated with FOLFOX at concentrations of 0.5 µM, 1 µM, 10 µM, 50 µM, 100 µM, and 200 µM. After 72 h of treatment, organoid viability was assessed using a cell viability assay. Each drug concentration for every organoid line was tested in at least three replicate wells.

### Cell viability assay

Cell viability was measured using the CellTiter-Glo 3D Cell Viability Assay (Promega) in white, flat bottom 96-well plates (Corning) according to the manufacturer’s instructions. The luminescence readout was performed using a CLARIOstar Plate Reader (BMG LabTech). Luminescence data were normalized by subtracting the background luminescence (positive control) from the experimental values and then scaling them relative to the untreated control (negative control), which was set to 100% viability. All the results were presented as relative cell viability (%). All experiments were conducted with at least three technical triplicates.

### RNA-seq analysis

The 3-prime RNA sequencing was processed using the published Drop-seq pipeline (v1.0) to generate sample- and gene-wise UMI Table [49]. The human reference genome GRCh38 derived from the Gencode homepage (EMBL-EBI) was used for alignment of sequences. Transcript and gene definitions were used according to the Gencode v.38 [50]. The data was processed in R using the DESeq2 package (v1.36) for the read normalization and variance stabilizing transformation [51]. Differential gene expression analysis was performed using the Wald test. Log_2_-fold changes were shrunken using the apeglm method to improve effect size estimation. Unless stated otherwise, a gene was considered significantly differentially expressed if the p-value was below 0.01 and the absolute apeglm-shrunken log_2_-fold change exceeded 0.5. Gene set variation analysis (GSVA) was performed on rlog normalized gene expression data using the GSVA r package (v1.52.3) [52]. Gene set libraries MSigDb-Hallmark-2020 of enrichR [53] were utilized for analyses. Gaussian kernel was used for non-parametric estimation of the cumulative distribution function of (sorted) expression levels, and normalized GSVA scores were extracted. Heatmaps were generated using ComplexHeatmap [54].

Differentially expressed genes were analyzed using STRING v.12 (Search Tool for the Retrieval of Interacting Genes/Proteins) [55]. Interactions were assessed based on known and predicted associations, including experimental data, co-expression, and curated pathway databases. A confidence score threshold of 0.4 (medium confidence) was applied to filter interactions and identify key regulatory hubs.

The CRC Intrinsic Subtypes (CRIS) were determined based on RNA-seq expression profiles using the CRISclassifier R package, as previously described [11]. Gene expression signatures were used to classify samples into one of the five CRIS subtypes. To ensure robust classification, the permutation parameter was set to 1,000.

### Copy number variant (CNV) analysis

Raw sequencing reads of the low-coverage whole genome sequencing (lcWGS) were trimmed using Trimmomatic (v0.39) [56], removing leading and trailing bases with Phred scores below 25 and reads with less than 50 nucleotides. In addition, an average base quality of 25 was enforced with a sliding window of 10 nucleotides for the reads. Passing reads were then aligned to the GRCm38.p6 reference genome using BWA-MEM (v0.7.17) [57] with default settings. The mapped reads were processed with samblaster (v0.1.26) [58], sambamba (v0.7.0) [59] and Picard tools (v2.20.0) (http://broadinstitute.g.ithub.io/picard). Copy number calling was performed with CNVKit [60] using the whole genome sequencing mode combined with the Agilent SureSelect Human All Exon Kit probe regions as on-target intervals, following CNVKit best-practice recommendations. The percentage of the genome altered (PGA) was calculated to assess the overall copy number alteration (CNA) burden at the genomic level (X and Y chromosomes were excluded). First, the total lengths of amplifications and deletions were determined, and their sum was divided by the total genome length to obtain the PGA values. Chromosome-wide CNVs were visualized using a heatmap generated with the CNVKit tool.

### Panel sequencing analysis

Raw sequencing data was processed using the Torrent Suite Software (version 5.2.2). Alignment against the human genome (version hg19) was performed using TMAP algorithm. Coverage data and variant calling was obtained using the build-in plugins “variantCaller” v5.8.0.19 and “coverageAnalysis.” Variant annotation was performed using a custom-built variant annotation pipeline based on ANNOVAR [61]. Visualization of sequencing reads was done using the Integrative Genomics Viewer Browser (IGV, http://www.broadinstitute.org/igv/). Variants were checked against the COSMIC (catalogue of somatic mutations in cancer) database [62], dbSNP, and Gnomad database [63], and only somatic mutations were included. Copy number variants (amplifications and deletions) were predicted using the coverage data summary for each sample and amplicons generated by the Torrent Suite software applying a four-step algorithm as previously described [64]. An amplification is predicted true when all amplicons covering a gene differs > 2 standard deviation from the median value. Deletions were reported if the standard deviation of all amplicons covering a gene is < 0.5.

### Microsatellite stability analysis

Microsatellite instability typing was performed using the marker panel *BAT25*, *BAT26*, *DSS346*, *D2S123* and *DI7S250* as previously described [65]. High microsatellite instability (MSI-H) was scored true if at least two of five markers showed genetic instability. Methylation-status of MLH-1 was determined using MethyQESD (methylation-quantification of endonuclease-resistant DNA) as described previously [66].

### Statistical analysis

All statistics and data visualization were performed in R (v4.1.2) and GraphPad Prism (v.10.1.1). Differences between groups were analyzed using a two-way analysis of variance (ANOVA) followed by Dunnett’s multiple comparisons test to compare each experimental condition to the respective control. Data are presented as mean ± standard error of the mean (SEM), unless stated otherwise. A statistical significance was considered when p-value < 0.05. No statistical method was used to pre-determine the sample size.

## Results

### Patient-derived organoids show specific morphologic patterns according to the histopathological CRC subtype and mirror the molecular profile of the primary tumor

**Fig. 1:**
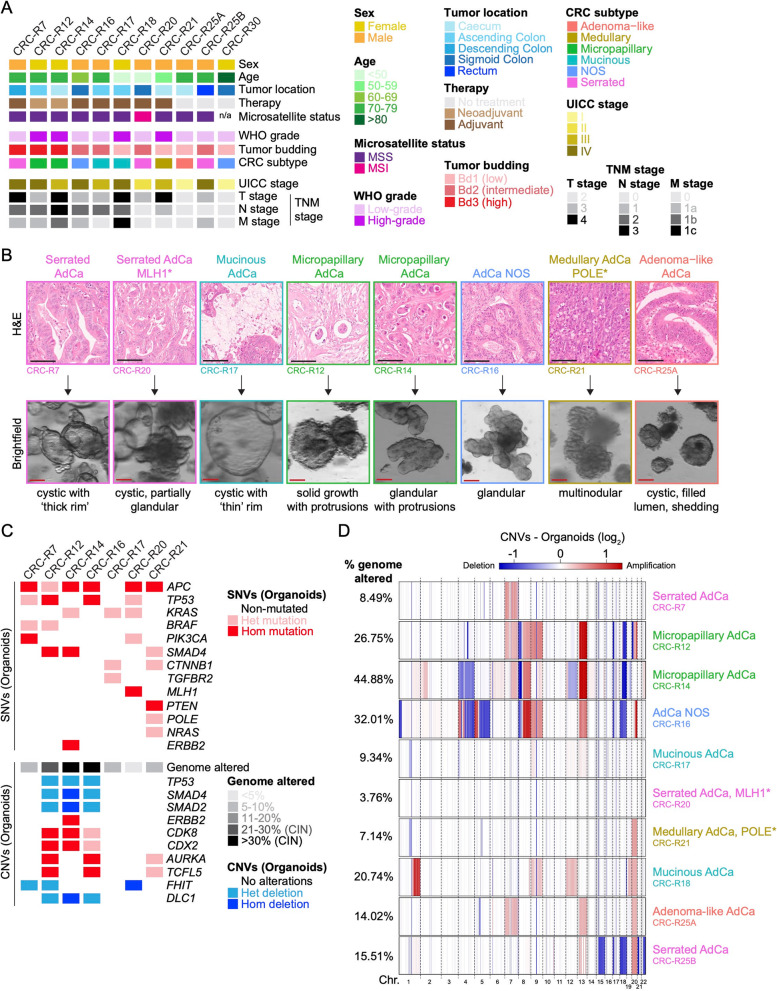
Patient-derived CRC organoids recapitulate histopathological and molecular tumor diversity. **A** Overview of the demographic (sex, age) and clinical characteristics (tumor location, therapy status, microsatellite status, WHO grade, tumor budding, CRC subtype, UICC and TNM stage) of patients included in the study (*n* = 11). CRC-Rx represents anonymized patient identifiers. **B** Representative brightfield and hematoxylin and eosin (H&E) stained images of PDOs displaying distinct histological CRC subtypes included in the study. Scale bars, 100 μm. **C** Molecular characterization of PDO samples selected for orthotopic transplantation (*n* = 7), analyzed by targeted panel sequencing and low-coverage whole-genome sequencing (lcWGS). The most frequent single nucleotide variants (SNVs) and copy number variants (CNVs) identified across the cohort are depicted. CIN, chromosomal instability. **D** Genome-wide CNV analysis displaying chromosomal amplifications (red) and deletions (blue) in individual chromosomes (Chr) of a subset of CRC PDOs (*n* = 10). The percentage of the genome affected by CNVs is indicated

To establish a robust preclinical model for CRC, we created a diverse biobank of patient-derived organoids (PDOs) representing the spectrum of CRC histology. The patients’ tumors were thoroughly characterized based on key morphological features defined by the WHO classification of digestive system tumors, including histological subtype, tumor budding activity, and WHO-grade [5]. The cohort included both right- and left-sided CRCs, spanning all UICC stages. Tumors from patients with diverse treatment backgrounds (treatment-naïve, neoadjuvant-treated, and adjuvant-treated) were included to ensure broad representativeness (Fig. 1A).

PDOs were successfully generated from the most common histopathological CRC subtypes (adenocarcinoma NOS, serrated adenocarcinoma, mucinous adenocarcinoma, micropapillary adenocarcinoma, medullary carcinoma, and adenoma-like adenocarcinoma), as well as from tumors with different grades and tumor budding categories (Fig. 1A). Upon differentiation in culture, PDOs exhibited distinct morphological growth patterns that closely reflected their histological subtypes. Serrated PDOs formed cystic structures with a thick rim and characteristic small invaginations and extravaginations along the surface. Mucinous PDOs appeared as thin-rimmed cysts with a largely hollow lumen. Micropapillary PDOs showed solid growth composed of small, rounded protruding cell clusters, forming a pseudopapillary, floret-like architecture. Adenocarcinoma NOS PDOs developed irregular glandular structures without a defined lumen. Medullary PDOs exhibited a multinodular, solid morphology with an irregular outer contour, consistent with the syncytial growth pattern seen in the original tumors (Fig. 1B).

Since PDOs represent the epithelial compartment of the tumor, they provide a stroma- and immune cell-free platform for precise genomic analysis. This enables the identification of tumor-specific single nucleotide variants (SNVs, mutations) without contamination from non-neoplastic cells. Genomic analysis identified key driver mutations consistent with known CRC profiles, including Wnt pathway activation (via *APC* loss or *CTNNB1* mutations), as well as activating mutations in *KRAS*, *BRAF*, and *PIK3CA*, and inactivating mutations in *TP53*, *SMAD4*, and *PTEN* (Fig. 1C and Table S1). In line with previous data, low-coverage whole genome sequencing (lcWGS) revealed that some organoids exhibited extensive chromosomal instability (CIN), while others maintained chromosomal stability (Fig. 1C-D). PDOs derived from micropapillary adenocarcinomas and adenocarcinoma NOS were classified as CIN tumors, whereas others remained chromosomally stable. As expected, hypermutated and ultra-hypermutated tumors, such as those harboring *MLH1* or *POLE* mutations, displayed minimal copy number alterations and a low percentage of the genome altered by structural variants (Fig. 1D). As a control, we also established organoids from low-grade tubular and tubulovillous adenomas (Fig. S1A). In line with their early-stage nature, adenomas exhibited no significant copy number alterations [67] and a low percentage of genome altered (Fig. S1B). We also analyzed common copy number variants (CNVs) associated with CRC development (Fig. 1C) [68]. Notably, we did not observe copy number losses in *CDKN2A*, *TGFBR2*, or *FBXW7*, nor copy number gains in *MYC*. Additionally, no CNV-driven alterations were detected in *RNF43*.

In summary, PDOs can be successfully established from diverse CRC subtypes, faithfully recapitulating their distinct morphological, molecular, and genomic features, providing a robust platform for precision oncology research.

### Subtype-specific histological features of patients’ tumors are retained in organoids after transplantation

**Fig. 2:**
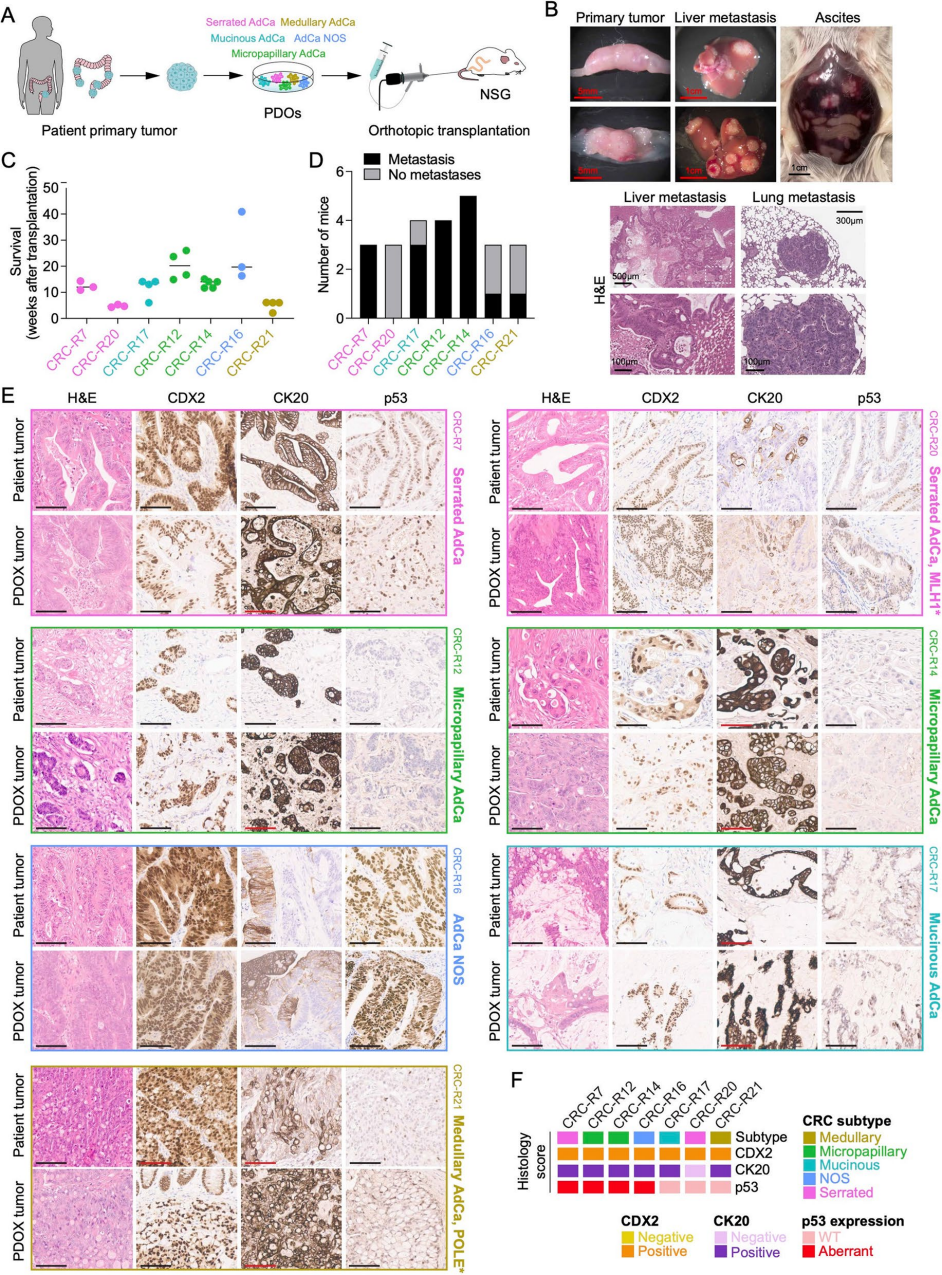
Orthotopic transplantation of human CRC-derived organoids results in colon tumor formation and metastasis, recapitulating patient tumor histology. **A** Schematic workflow of the orthotopic CRC mouse model, depicting the isolation of tumor cells from CRC patients representing different histological subtypes, followed by organoid generation and subsequent transplantation into NSG mice. **B** Representative images showing the localization of primary tumors in the colon of recipient mice following orthotopic transplantation of CRC organoids. Metastatic spread to the liver and the presence of ascites were frequently observed, consistent with common CRC metastatic routes. Size of scale bars are indicated in the image. **C** Bar plot displaying survival times (in weeks) of recipient mice (*n* = 26) following orthotopic transplantation of distinct PDOs. On average, 3–5 mice were transplanted per organoid line. **D** Histogram quantifying the number of mice that developed metastases after orthotopic transplantation, stratified by the transplanted PDO line in **C**. **E** Representative hematoxylin and eosin (H&E) staining and immunohistochemistry for CDX2, CK20, and p53 in PDOX tumors. Marker expression and histological features in PDOX tumors closely resemble those of the corresponding patient tumors (*n* = 7). Scale bars, 100 μm. **F** Histopathological classification of patient tumors used to establish PDOXs, based on H&E staining and the expression of CDX2, CK20, and p53 (*n* = 7) in **E**

To establish a controllable in vivo model for CRC, we orthotopically transplanted PDOs into the distal colon and rectum of immunodeficient NSG mice via colonoscopy-guided submucosal injections, as previously described (Fig. 2A) [39, 42]. We transplanted seven distinct CRC PDO lines into 28 NSG mice. Successful engraftment occurred in 26 mice, leading to the formation of PDOXs that developed tumors at the injection sites, with stable survival rates supporting the feasibility and robustness of the orthotopic organoid transplantation model (Fig. 2B-C). In some cases, distant metastases were observed in organs such as the liver and lungs (Fig. 2B and D). Moreover, three mice developed malignant ascites at necropsy (Fig. 2B).

Notably, adenoma-derived organoids did not form tumors upon transplantation (data not shown), likely due to a failure to establish a proliferative niche or to undergo malignant transformation, consistent with previous observations in healthy tissue-derived organoids [69].

Importantly, all PDOX tumors retained their key histological features observed in the respective patient tumor, including histological subtype, tumor budding activity, and tumor grade, despite the absence of a human-derived stromal compartment. Furthermore, common CRC markers (CDX2/CK20) and p53 showed consistent expression patterns between PDOX tumors and the respective patient primary tumors (Fig. 2E-F). A comparative analysis of epithelial cytoskeletal organization (E-cadherin), cell-cell junctions (Desmoplakin and Claudin-1), and stromal content (Masson-Goldner trichrome) was performed in seven matched pairs of PDOX tumors and the corresponding patient primary tumors (Fig. S2A-D). All markers were consistently expressed, with E-cadherin and Claudin-1 showing concordant, strong, and diffuse staining across human and murine tumor components, indicating preserved epithelial architecture and intact adherens and tight junction-associated differentiation. Desmoplakin expression was broadly comparable, showing moderate to strong staining with minor inter-case variability. Stromal content was largely similar between human and PDOX samples, predominantly moderate, with only minor variation (Fig. S2A-D).

**Fig. 3:**
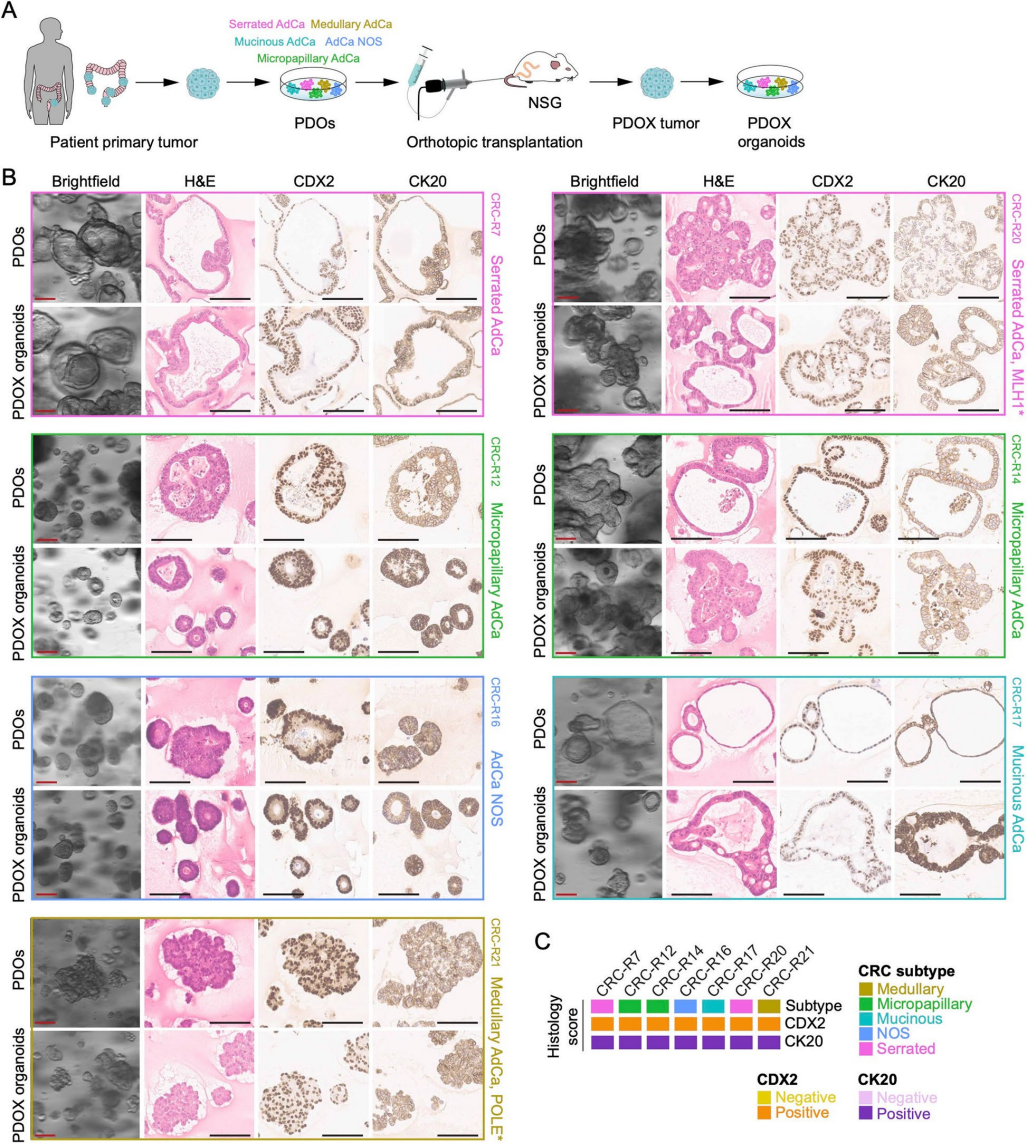
Mouse PDOX-derived organoids retain morphology, histology, and marker expression of PDOs. **A** Schematic workflow of the orthotopic CRC mouse model, illustrating the isolation of tumor cells from PDOX tumors and the subsequent generation of PDOX-derived organoids. **B** Representative images of brightfield microscopy, H&E staining, and immunohistochemical staining for CDX2 and CK20 in seven distinct PDO-PDOX organoid pairs embedded in paraffin (FFPE), representing all histological subtypes included in the study. **C** Histological classification based on H&E staining and evaluation of CDX2 and CK20 marker expression in PDOs and their corresponding PDOX-derived organoids shown in (B) (*n* = 7)

After confirming the histotypes of the PDOX tumors, we aimed to determine whether their genomic and molecular characteristics, including CRC subtype-specific features, were retained. To this end, we successfully established 32 PDOX-derived (secondary) organoid cultures, including 22 from primary PDOX tumors and 10 from metastatic sites (Fig. 3A). Remarkably, these organoids retained the distinct morphology observed in their corresponding PDOs (Fig. 3B-C). Additional histological sections and immunohistochemical evaluation confirmed a consistent H&E morphology and CRC marker profile across PDOs, PDOX-tumor-derived organoids, and their metastatic counterparts.

In summary, our findings demonstrate that PDOX carcinomas faithfully retain the histopathological characteristics of the corresponding patient tumors with minimal variation. Moreover, PDOX-derived secondary organoids remained morphologically stable and closely resembled primary PDOs.

### Genomic and transcriptomic stability of patient-derived CRC organoids and PDOX models

**Fig. 4:**
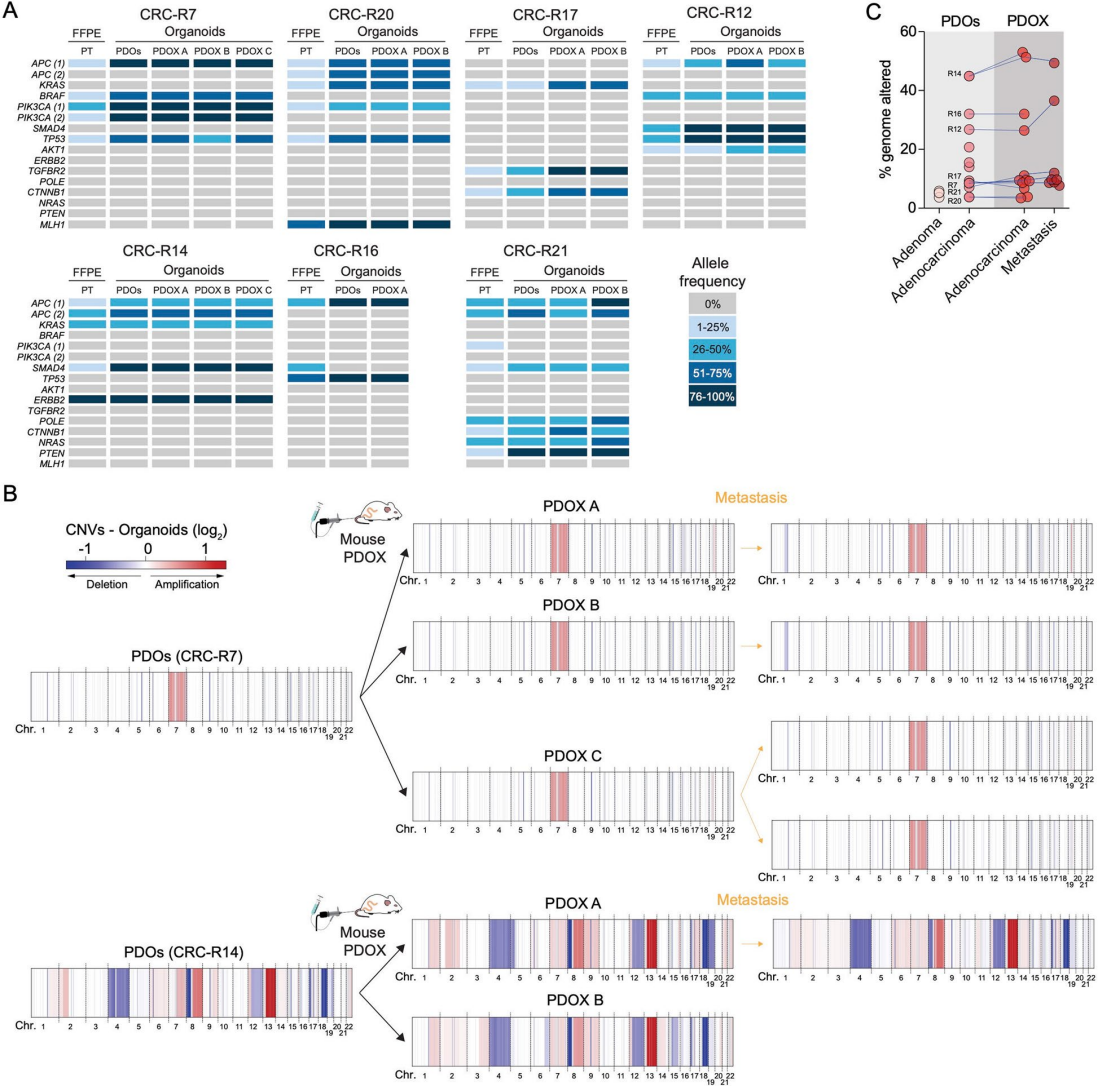
PDOX-derived organoids recapitulate key genetic and molecular features of both human tumor tissue and PDOs. **A** Overview of the most common SNVs and their respective allelic frequencies across different sample types, including patient tumor tissue (FFPE) and organoids (PDOs and PDOX-derived organoids) for each of the human CRC samples used to generate PDOXs (*n* = 7). PT, patient tumor. **B** Comparative analysis of CNV profiles between PDOs and PDOX-derived organoids, including primary mouse tumors and metastases, for two representative human CRC samples. CNV profiles of additional PDO-PDOX pairs are shown in Fig. S4. **C** Quantification of the percentage of genome altered in individual organoid samples before (PDOs) and after (PDOX) orthotopic transplantation. Adenoma and additional adenocarcinoma organoids were included in this analysis

To investigate whether genomic features remain stable or undergo evolutionary progression across their different states, from patient tumors to PDOX-derived organoids, we conducted targeted panel sequencing on (i) FFPE patient tumor samples, (ii) PDOs, and (iii) organoids derived from PDOX models. Overall, the genetic landscape of the PDOX-derived organoids closely mirrored that of the respective patient tumors, with only minor additional alterations and variations in mutated allele frequencies (Fig. 4A). Notably, we observed an average increase of 31.77% in the allele frequency of tumor-specific mutations from FFPE tumor samples to PDOs, which can be attributed to the absence of stromal contamination in organoid cultures (Fig. S3A). Furthermore, the allele frequency remained highly stable from PDO to PDOX, with an average increase of 7.55%, indicating that organoid models faithfully preserve the genetic characteristics of the original tumor during in vivo transplantation (Fig. 4A and Fig. S3B).

Next, we performed lcWGS of PDOX-derived tumor and metastasis organoids. Our analysis revealed no additional chromosomal deletions or amplifications during organoid culture or subsequent transplantation and tumor growth in mice (Fig. 4B and Fig. S4A). This suggests minimal genomic evolution when organoids are maintained under optimal conditions, free from selective pressures. Consistently, the percentages of genome altered before and after transplantation remained largely stable, with changes ranging from − 2.61% to + 8.12% (average increase of + 1.51%) in PDOX tumor organoids and − 1.64% to + 9.74% (average increase of + 2.53%) in PDOX metastasis organoids. This supports the idea that tumor subtypes maintain their genomic identity in vivo (Fig. 4C).

**Fig. 5:**
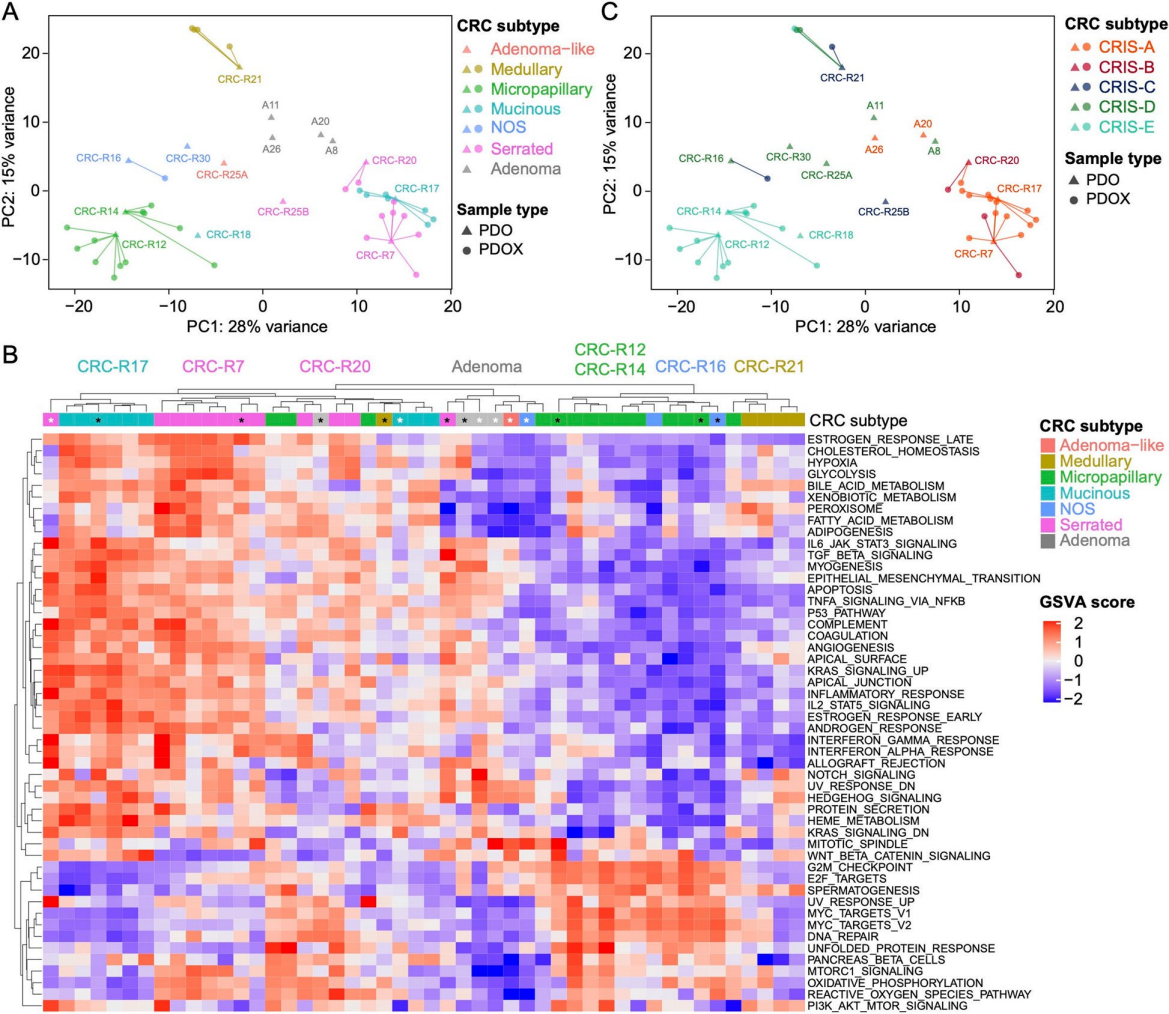
Transcriptomic profiling enables subtype-specific clustering of CRC-derived organoids. **A** Principal component analysis (PCA) illustrating the clustering of samples based on histological subtype. The lines connecting the points indicate the relationship between each PDO and its corresponding PDOX models. Samples are color-coded by CRC subtype, and point shapes distinguish between PDOs and PDOXs. **B** Heatmap of gene set variation analysis (GSVA) depicting distinct pathway activation across different clusters. Hallmark pathway enrichment analysis highlights unique biological processes associated with each cluster, with color intensity representing the degree of enrichment (red) and reduction (blue). Black stars indicate PDOs used for transplantation. White stars indicate PDOs not used for transplantation. **C** PCA demonstrating the clustering of samples according to the CRIS subtypes. The lines connecting the points indicate the relationship between each PDO and its corresponding PDOX models. Samples are color-coded by CRC CRIS classification, and point shapes distinguish between PDOs and PDOXs

To further assess molecular consistency between PDOs and PDOX-derived organoids, we conducted RNA sequencing and performed unsupervised clustering. Importantly, mouse-derived organoids clustered with their respective PDOs, confirming the retention of molecular identity post-transplantation (Fig. 5A and Fig. S5A). Notably, the different histopathological CRC subtypes also clustered accordingly. Mucinous and serrated adenocarcinomas, as well as micropapillary and adenocarcinoma NOS formed distinct clusters (Fig. 5A). Additionally, while the adenoma-like adenocarcinoma line CRC-R25A clustered with the four adenoma organoid lines, the medullary adenocarcinoma organoid line formed a distinct cluster along the PC2 axis (Fig. 5A).

To gain deeper insights into the transcriptomic landscape, we performed gene set variation analysis (GSVA), which further supported the clustering observed in the Principal component analysis (PCA) (Fig. 5B). Specifically, PDOs and PDOX-derived organoids from mucinous and serrated adenocarcinomas exhibited high activation of hallmark cancer-related pathways, including epithelial-mesenchymal transition (EMT), IL-6-JAK-STAT3 signaling, TGF-β signaling, KRAS activation, and p53 signaling. These subtypes also displayed enrichment in inflammatory and immunological pathways (e.g., TNF-α signaling via NF-κB, interferon-α and -γ response) as well as metabolic pathways (glycolysis, bile acid metabolism, fatty acid metabolism, cholesterol homeostasis, and xenobiotic metabolism). Notably, protein-protein interaction analysis using STRING revealed an enrichment of mucin-related genes, alongside inflammatory and immunological pathways, in mucinous adenocarcinoma organoids (cluster 3; Fig. S5B). In contrast, micropapillary and adenocarcinoma NOS subtypes exhibited upregulation of pathways associated with proliferation and cell cycle regulation (E2F targets, G2M checkpoint, MYC targets v1 and v2), along with DNA damage response pathways (DNA repair, UV response, reactive oxygen species pathway, oxidative phosphorylation, and unfolded protein response) (Fig. 5B). STRING analysis revealed an enrichment of UGT1A and UGT2B enzymes in micropapillary adenocarcinoma organoids (cluster 2; Fig. S5B). These enzymes, part of the UDP-glucuronosyltransferase (UGT) family, are involved in phase II drug metabolism and detoxification processes, while also contributing to oxidative stress regulation and ROS homeostasis [70], suggesting a potential role in the unique metabolic landscape of this CRC subtype. This indicates that mucinous and serrated adenocarcinomas have distinct transcriptional programs compared to micropapillary adenocarcinoma and adenocarcinoma NOS. Medullary adenocarcinoma clustered separately in the PCA plot, but their signatures were partially overlapping with those from micropapillary and adenocarcinoma NOS. Specifically, we observed a pronunciation of immune-related genes such as TNF, IL-6 or IL-10, which is consistent with the usually highly immunogenic nature of the medullary subtype (Fig. 5B and Fig. S5B) [71–73].

Next, we applied the CRC intrinsic subtyping (CRIS) classification, which was specifically designed for in vitro models lacking immune and stromal components (see Methods for details) [11]. The CRIS subtypes are defined as follows: (i) CRIS-A: Mucinous, glycolytic, enriched for microsatellite instability (MSI) or *KRAS* mutations; (ii) CRIS-B: TGF-β pathway activation, EMT, associated with poor prognosis; (iii) CRIS-C: Elevated EGFR signaling, sensitivity to EGFR inhibitors; (iv) CRIS-D: WNT activation, IGF2 overexpression and amplification; (v) CRIS-E: Paneth cell-like phenotype, *TP53* mutations. Applying this classification, we observed that all CRIS subtypes were represented within our organoid lines (Fig. 5C). Strikingly, the transcriptomic clustering of the histopathological CRC subtypes correlated well with CRIS classification, reinforcing the robustness of this subtyping approach. The mucinous adenocarcinoma line (CRC-R17) and its PDOX-derived counterparts were consistently classified as CRIS-A, aligning with their metabolic and inflammatory Hallmark pathway activation. Similarly, the serrated adenocarcinoma line CRC-R7 was categorized as CRIS-A, though some PDOX derivative lines switched to CRIS-B, reflecting their TGF-β activation and EMT properties. Additionally, CRC-R20 was initially classified as CRIS-B, with one PDOX organoid line transitioning to CRIS-A. Interestingly, one non-transplanted serrated adenocarcinoma line (CRC-R25B) was classified as CRIS-C. Among adenoma lines, A20 and A26 were classified as CRIS-A, while A8 and A11 were classified as CRIS-D. The micropapillary adenocarcinoma organoid lines (CRC-R12 and CRC-R14), along with their PDOX-derived counterparts, consistently mapped to CRIS-E. The adenocarcinoma NOS lines (CRC-R16 and CRC-R30) were classified as CRIS-D. On the other hand, while the medullary adenocarcinoma line (CRC-R21) was initially classified as CRIS-C, though most of its PDOX-derived lines transitioned to CRIS-D or CRIS-E post-transplantation (Fig. 5C), suggesting plasticity in this subtype that was also evident in the heatmap of the GSVA (Fig. 5B).

In summary, our data demonstrate that the mutational landscape, copy number alterations and transcriptomic profiles of patient tumors are faithfully retained in PDOs and remain stable following orthotopic transplantation. Furthermore, our findings reveal a strong alignment between CRIS classification and transcriptomic Hallmark pathway activation, demonstrating that CRC subtypes retain distinct molecular features in PDO and PDOX models.

### Phenotypic stability and chemosensitivity of PDO and PDOX models in response to FOLFOX treatment

To assess the fidelity of PDOs and their corresponding PDOX models in response to chemotherapy, we initially tested three representative CRC PDO lines reflecting distinct treatment backgrounds. These included one derived from a patient who had received adjuvant FOLFOX therapy (CRC-R7, serrated adenocarcinoma), one from a patient who underwent neoadjuvant FOLFOX treatment (CRC-R12, micropapillary adenocarcinoma), and one from a chemo-naïve patient (CRC-R20, serrated adenocarcinoma, MSI). This selection aimed to capture the variability in treatment history and its potential impact on drug response.

In preliminary experiments, PDOs were treated with FOLFOX at increasing concentrations (1 µM, 10 µM, and 50 µM) for 24 h, 48 h, and 72 h (Fig. S6A). PDOs derived from chemo-naïve patients (CRC-R7, CRC-R20) displayed marked sensitivity to FOLFOX, with complete growth inhibition at concentrations of 10 µM and 50 µM. In contrast, PDOs from the neoadjuvantly treated patient (CRC-R12) maintained their growth and viability, suggesting the presence of pre-existing resistance mechanisms. When PDOX-derived organoids from these three models were treated with 50 µM FOLFOX (Fig. S6B), all PDOX lines exhibited identical treatment responses to their corresponding PDOs, reinforcing that these models faithfully preserve not only histomorphological, molecular, and genomic characteristics but also patient-specific drug responses.

**Fig. 6:**
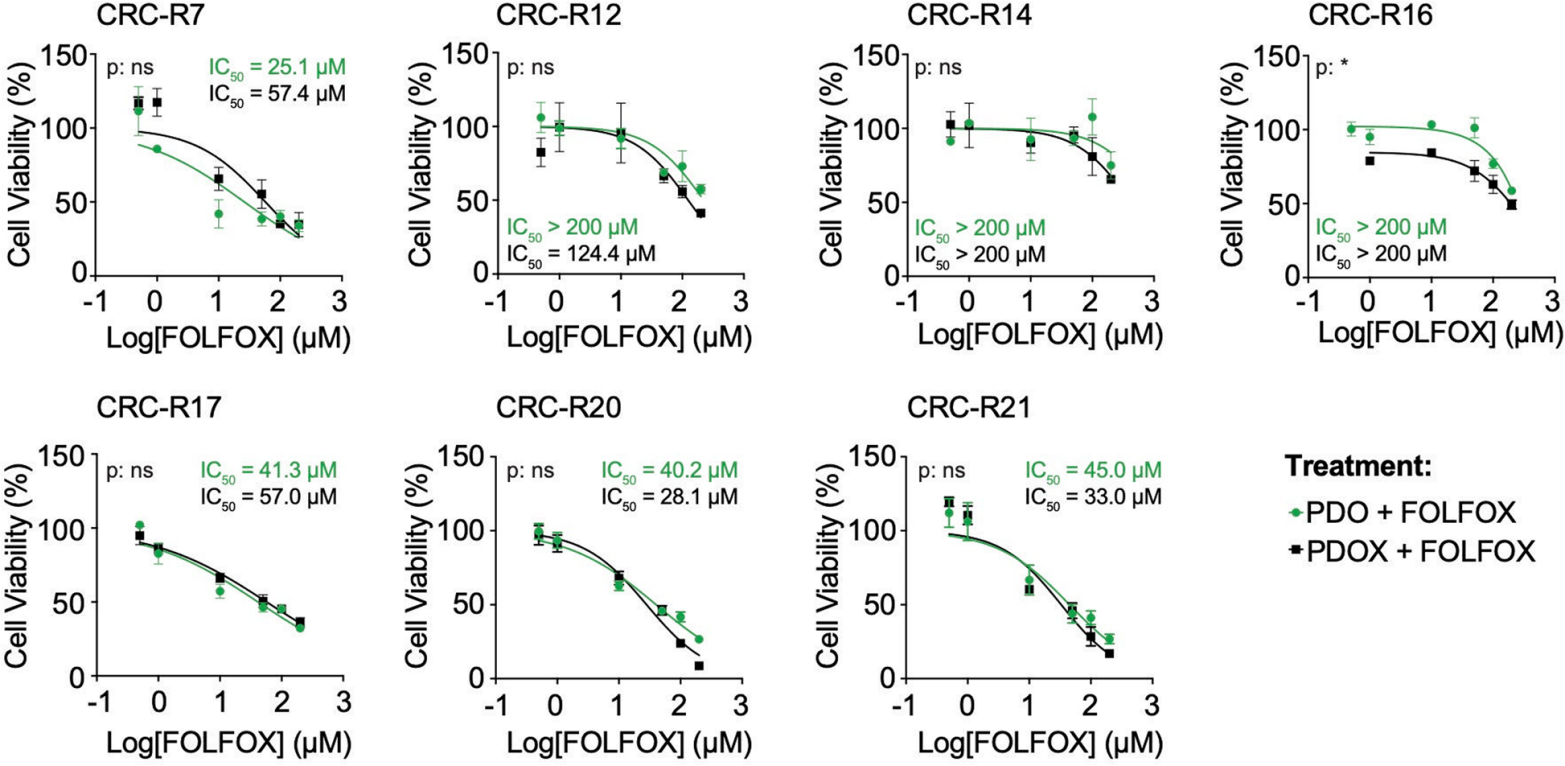
PDOX-derived organoids exhibit similar drug responses to FOLFOX treatment as PDOs. Cell viability of PDOs and their matched PDOX-derived organoids was assessed after 72 h of treatment with increasing concentrations of FOLFOX (0.5 µM, 1 µM, 10 µM, 50 µM, 100 µM, and 200 µM) using the CellTiter-Glo^®^ Luminescent Cell Viability Assay. Relative luminescence units (RLU) were normalized to untreated controls. Each data point represents the mean ± SEM of three technical replicates. Dose-response curves were fitted using a nonlinear regression model (log[FOLFOX] vs. response, variable slope), and half-maximal inhibitory concentrations (IC_50_) were determined. Statistical differences between curves were evaluated using the extra sum-of-squares F-test. Data are presented as mean ± SEM. Ns, non-significant; * *p* < 0.05

To comprehensively validate these findings, we expanded the analysis to include all PDO lines used in this study and their matched PDOX models (Fig. 6). Organoids were treated with a broader range of FOLFOX concentrations (0.5 µM, 1 µM, 10 µM, 50 µM, 100 µM, and 200 µM) for 72 h, followed by assessment of cell viability. Consistent with the preliminary data, PDOX lines closely mirrored the drug responses of their corresponding PDOs.

Three models, CRC-R12, CRC-R14, and CRC-R16, exhibited pronounced resistance to FOLFOX, with IC_50_ values exceeding the highest tested concentration (200 µM) (Fig. 6). CRC-R12 and CRC-R16 harbored homozygous *TP53* mutations (Table S1). CRC-R12 and CRC-R14 originated from patients who had received neoadjuvant FOLFOX and CAPOX therapy, respectively, whereas CRC-R16 was derived from a tumor resected prior to adjuvant FOLFOX treatment and therefore had not been exposed to chemotherapy. The resistance observed in these lines likely reflects therapy-driven selection in CRC-R14, *TP53* deficiency-mediated resistance in CRC-R16, and a combination of both mechanisms in CRC-R12. Loss of p53 function impairs DNA damage-induced apoptosis, while prior oxaliplatin/5-FU exposure can select for clones with reduced drug susceptibility, jointly contributing to the development of chemoresistance [74–76]. In contrast, PDOs and PDOX lines derived from untreated patients (CRC-R7, CRC-R17, CRC-R20, and CRC-R21) remained highly sensitive to FOLFOX, with IC_50_ values between 25 µM and 57µM.

These results demonstrate that PDO and PDOX models faithfully mirror patient-specific treatment histories and intrinsic genetic determinants such as p53 loss, providing functionally stable systems for predicting therapeutic responses in CRC.

## Discussion

The marked inter- and intratumoral morphomolecular heterogeneity of CRC results in a distinct histological and molecular profile for each patient, making it difficult to accurately predict disease progression and treatment response [77, 78]. Patient-derived models such as organoids (PDOs) and patient-derived xenografts (PDXs) have become valuable tools for studying CRC biology and therapy response [33]. However, PDOs alone are limited in their ability to reflect complex clinical behaviors due to their in vitro nature. In addition, little is known about how their morphology and molecular features change in a living environment.

Our study addresses this gap by providing an in-depth morphomolecular analysis of an orthotopic human organoid transplantation model. We show that both PDO and PDOX organoids closely mirror key histological, genomic, and transcriptomic features of the original patient tumors. We also generated second-generation organoids from these in vivo tumors and observed that essential morphological characteristics remained remarkably stable, along with the preservation of key molecular alterations. In exploratory drug screening assays, we observed consistent treatment responses across PDOs, PDOXs, and corresponding patient tissue. To our knowledge, this is the first study to systematically assess the phenotypic stability of PDO-based models across histological, genomic, and transcriptomic levels, both in vitro and after in vivo passaging.

Our findings align with previous studies demonstrating that CRC organoids maintain their mutational and transcriptomic landscapes across multiple in vitro passages [19, 22]. However, few studies have addressed whether PDOs retain these molecular profiles after in vivo transplantation [24, 33], and it remains unclear to what extent PDOs are capable of recapitulating the histological architecture of the primary tumor in a living environment. Our study is the first to comprehensively assess the histological and molecular stability of CRC PDOs after orthotopic transplantation, highlighting their potential as biologically accurate replicas of the primary disease.

In a first step, our comparative histological analysis revealed a remarkable consistency between the patient tumors and the corresponding PDOX tumors in mice, particularly with respect to the key morphological features of CRC. This stability encompassed not only the general histological subtype but also other architectural parameters, including tumor grade and tumor budding, the latter serving as a surrogate marker of dissociative growth behavior, as well as highly similar expression profiles of markers associated with epithelial cytoskeletal organization, cell-cell junction integrity, and stromal content.

Based on these observations of pronounced morphological stability, we next conducted a comparative molecular analysis by systematically characterizing organoids rederived from PDOX tumors, an approach not previously reported in CRC or other tumor types. Notably, both PDOX tumors and second-generation organoids retained their genetic integrity, with no evidence of additional chromosomal aberrations during in vivo passaging. This contrasts with reports of genomic drift in conventional PDX models over serial transplantations [14, 18] and underscores the advantage of organoid-derived xenografts in preserving genomic stability while allowing for long-term biobanking.

At the transcriptomic level, PDOs and PDOX-derived organoids clustered consistently and retained subtype-associated pathway activation, including CRIS classifications. Recent high-resolution analyses of PDXs, together with primary CRC datasets, have shown that tumors often harbor overlapping transcriptional programs rather than occupying a single discrete subtype, reflecting the concomitant presence of cells assigned to different CRIS classes or, less frequently, hybrid phenotypic states within the same lesion [79]. In this context, the stability observed in our bulk transcriptomic analyses likely reflects robust maintenance of the dominant transcriptional programs that define tumor identity, while not excluding he coexistence of additional biologically relevant states that may not be fully resolved at bulk resolution. Thus, our data support the notion that PDO/PDOX models preserve the core transcriptional architecture of CRC while remaining compatible with underlying biological heterogeneity.

In addition, our study reinforces the clinical relevance of PDOs as potentially predictive models for therapy response, as PDOs mirrored the clinical outcome of their respective patients in vitro. PDOs derived from chemo-naïve or adjuvantly treated patients exhibited sensitivity to FOLFOX, whereas PDOs from patients who had received neoadjuvant chemotherapy or harbored a homozygous *TP53* mutation displayed resistance. This resistance may reflect both prior treatment-induced clonal selection and the impaired DNA damage-induced apoptotic response characteristic of p53-deficient cells, which allows them to survive FOLFOX-induced genotoxic stress [74–76]. Recent systematic analyses across large CRC PDX and PDX-derived tumor organoid collections have demonstrated that resistance to 5-FU-based chemotherapy is governed by recurrent, functionally defined molecular programs rather than by idiosyncratic genetic alterations, and that these programs can be reproducibly maintained across patient-derived model systems [80]. In line with these observations, PDOXs derived from our resistant and sensitive lines maintained their respective response profiles in vivo, underscoring the capacity of PDO/PDOX systems to capture both durable treatment sensitivities and established resistance states. However, given the limited number of cases and the use of a single treatment regimen, these findings should be considered exploratory and warrant further investigation.

Our study has several limitations. First, the number of patients included was limited, with some histological subtypes represented by only a single PDO line. However, as an exploratory analysis, this study aimed to capture the most common histological variants of CRC and to assess the general concept of histological stability in PDO-based models. Large-scale CRC model resources have recently demonstrated the value of broad, population-level coverage for identifying common molecular and pharmacological patterns and for benchmarking therapeutic response across genetically diverse tumors [81]. Notably, resources such as the XENTURION platform integrate extensive collections of PDXs and matched PDX-derived tumor organoids with multidimensional molecular and drug response profiling, providing an important foundation for population-scale preclinical discovery. In contrast, our work prioritizes intensive longitudinal and cross-context characterization of a limited number of models, enabling detailed assessment of histological, genomic, transcriptomic, and functional stability across in vitro and in vivo settings. In this regard, our study represents a high-resolution, proof-of-concept analysis that illustrates how deeply annotated PDO/PDOX systems can deliver mechanistic insight and experimental tractability that complement the breadth of large-scale CRC model resources. Second, only a single chemotherapeutic regimen (FOLFOX) was tested, limiting the scope for conclusions regarding responses to other therapy regimens, targeted agents or immunotherapies – aspects that should be addressed in future studies. Furthermore, PDOX models were generated in immunodeficient NSG mice, restricting the ability to study tumor-immune system interactions. Therefore, we cannot exclude the possibility that selection pressures introduced by the in vivo environment may have influenced the observed tumor characteristics.

In summary, our study provides a comprehensive morphomolecular characterization of PDOs and PDOXs in comparison to the original patient tumors within the in vivo context of an orthotopic transplantation model, demonstrating their phenotypic and molecular stability as well as their predictive potential. We contribute novel insights into how well these models replicate patient tumors, supporting their application in both basic and translational CRC research. Future studies should explore how additional components of the tumor microenvironment influence PDOX behavior and assess whether the integration of immune and stromal elements could further improve how well these models capture tumor biology and support clinically meaningful applications.

## Conclusion

In this study, we established a comprehensive biobank of patient-derived CRC organoids encompassing major histological subtypes, key genomic alterations, and transcriptomic consensus molecular subtypes (CMS). Through extensive multi-omic characterization, we demonstrate that these organoids faithfully preserve the molecular and morphological diversity of patient tumors. Orthotopic transplantation into immunodeficient mice further validated the robustness of this model, as xenograft tumors maintained the subtype-specific histology and molecular signatures of their parental organoids. Remarkably, organoids re-derived from xenografts retained their original phenotypic and genomic features and exhibited similar responses to FOLFOX treatment, confirming the stability and fidelity of this platform. Together, our work establishes a deeply characterized and translationally relevant organoid resource that bridges histopathology, molecular profiling, and functional modeling, providing a powerful tool for dissecting CRC heterogeneity and advancing precision therapy development.

## Supplementary Information

Supplementary Material 1.

Supplementary Material 2.

## Data Availability

Low-coverage whole genome sequencing and 3-prime RNA sequencing data generated in this study are deposited under ENA accession number PRJEB107051. This paper does not report original code.

## Materials availability

All unique resources generated in this study are available from the lead contact with a completed materials transfer agreement.

## Abbreviations

CNV: Copy number variants
CMS: Consensus Molecular Subtypes
CRC: Colorectal cancer
CRIS: CRC Intrinsic Subtypes
EMT: Epithelial-mesenchymal transition
FFPE: Formalin-fixed paraffin embedded
FOLFOX: Folinic acid, Fluorouracil, and Oxaliplatin
GSVA: Gene set variation analysis
H&E: Hematoxylin & Eosin
IC50: Half-maximal Inhibitory Concentration
IRS: Immunoreactivity score
LcWGS: Low-coverage whole genome sequencing
NOS: Not otherwise specified
NSG: NOD scid gamma
PCA: Principal component analysis
PDO: Patient-derived organoids
PDOX: Patient-derived organoid xenografts
PDX: Patient-derived xenografts
SNV: Single nucleotide variants
SPF: Specific-pathogen-free
VAF: Variant Allele Frequency
