# Supplementary figures and images for: A translational colorectal cancer organoid biobank mirrors patients’ tumor histology, molecular profiles, and treatment responses
Source: J Exp Clin Cancer Res. 2026 Feb 25;45:89. doi: 10.1186/s13046-026-03666-x (PMC13059402; doi:10.1186/s13046-026-03666-x)

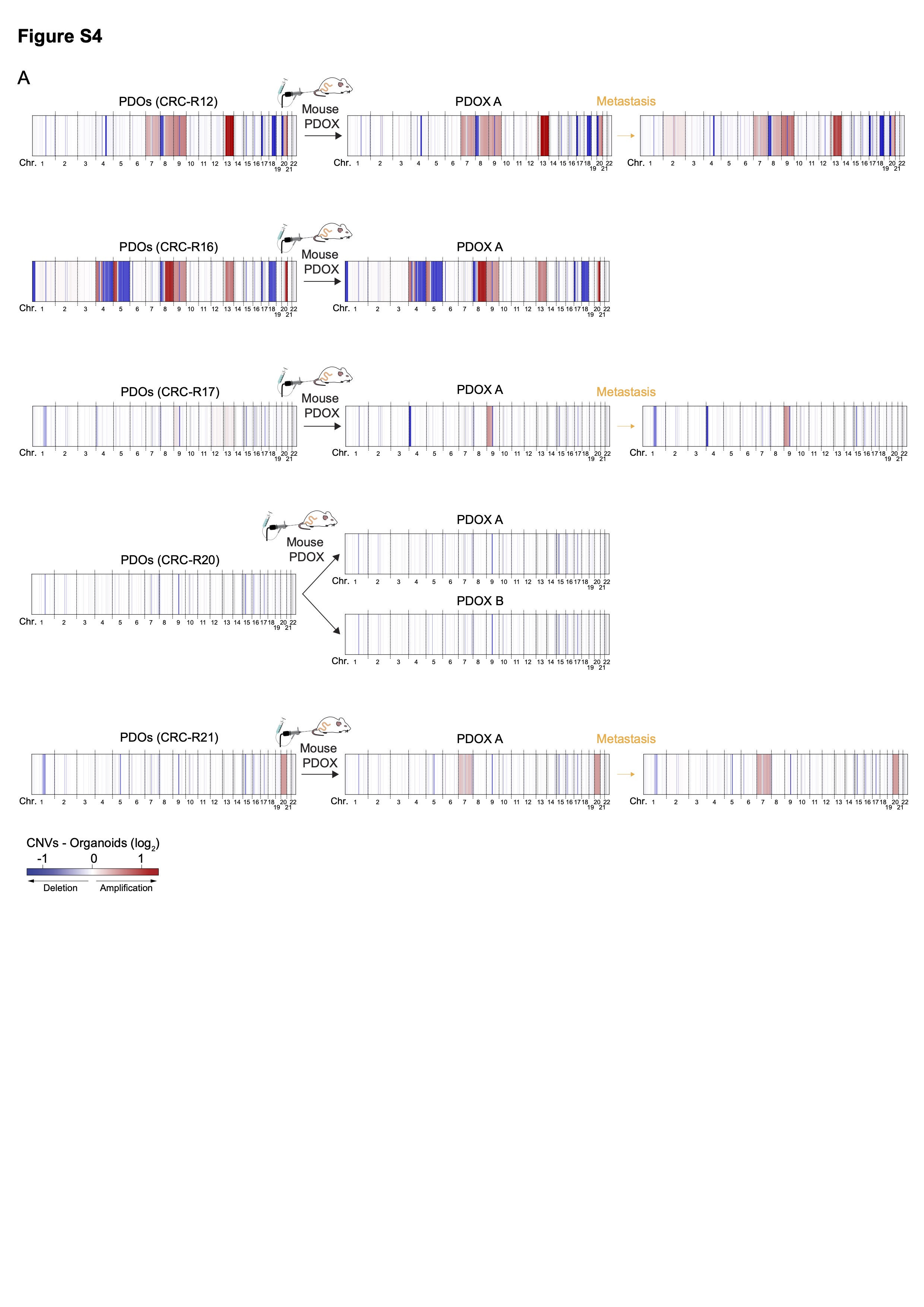

Supplement: Supplementary file 1 — Supplementary Material 1. [file 13046_2026_3666_MOESM1_ESM.zip › Figure S4.tiff]

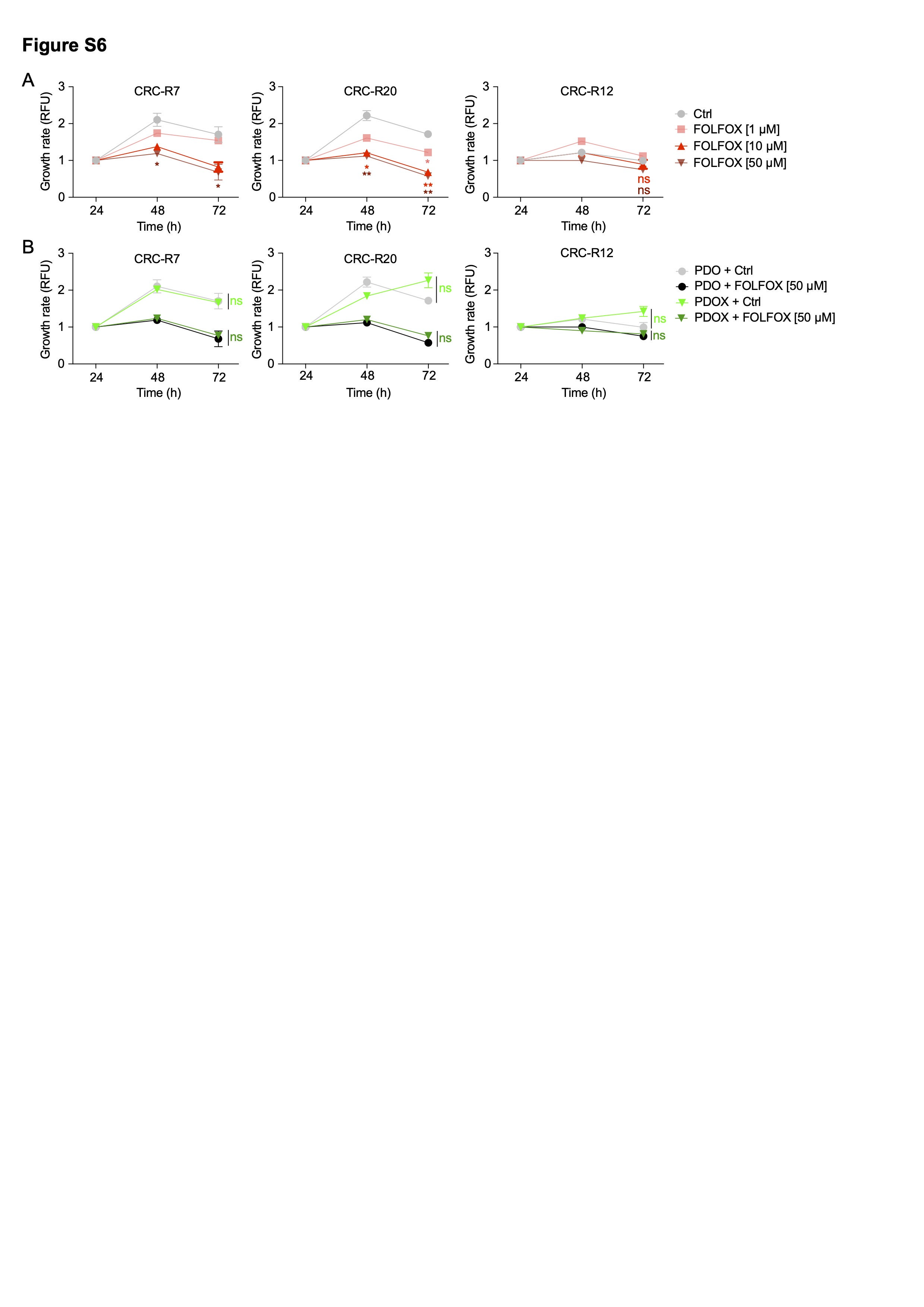

Supplement: Supplementary file 1 — Supplementary Material 1. [file 13046_2026_3666_MOESM1_ESM.zip › Figure S6.tiff]

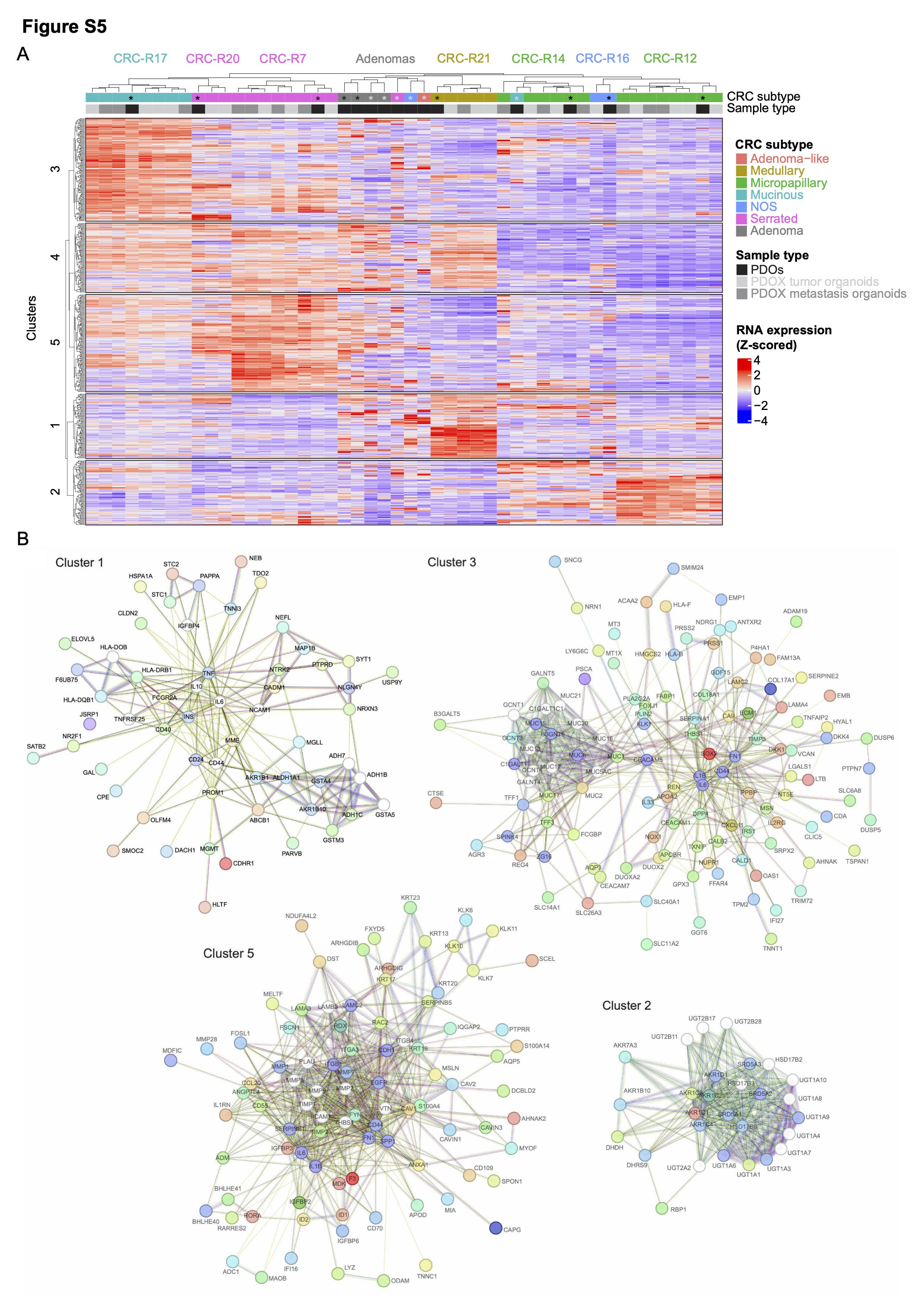

Supplement: Supplementary file 1 — Supplementary Material 1. [file 13046_2026_3666_MOESM1_ESM.zip › Figure S5.tiff]

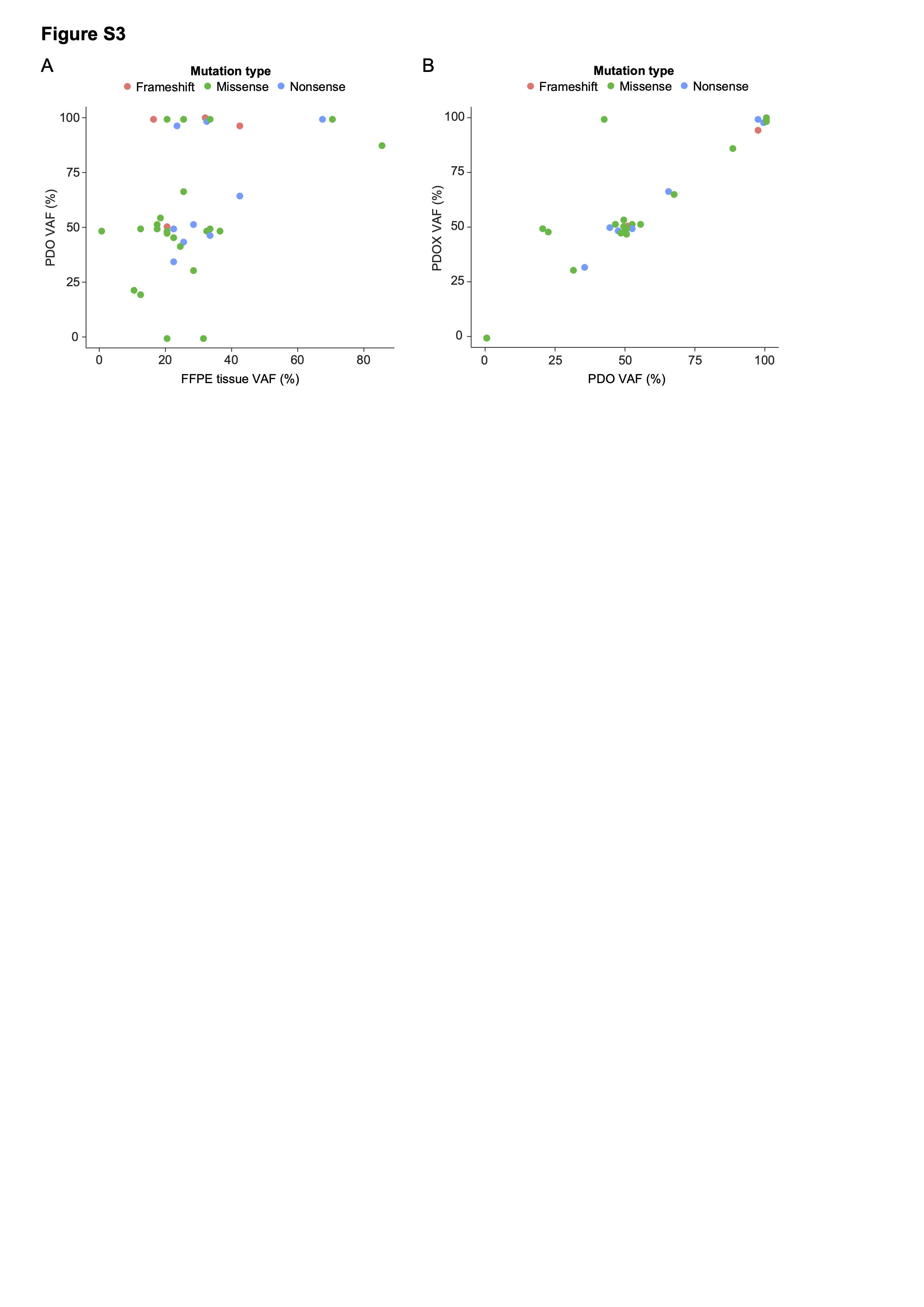

Supplement: Supplementary file 1 — Supplementary Material 1. [file 13046_2026_3666_MOESM1_ESM.zip › Figure S3.tiff]

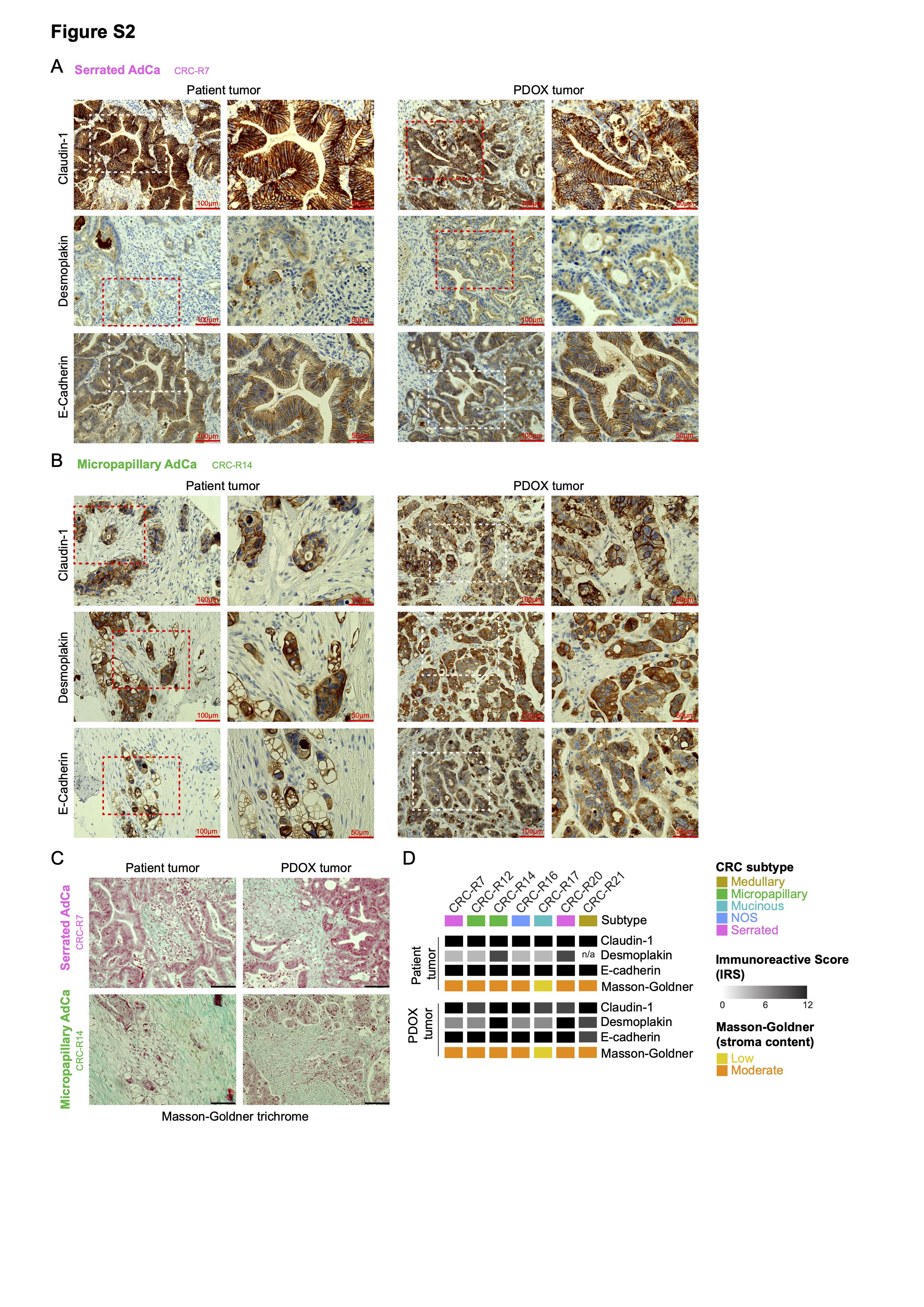

Supplement: Supplementary file 1 — Supplementary Material 1. [file 13046_2026_3666_MOESM1_ESM.zip › Figure S2.tiff]

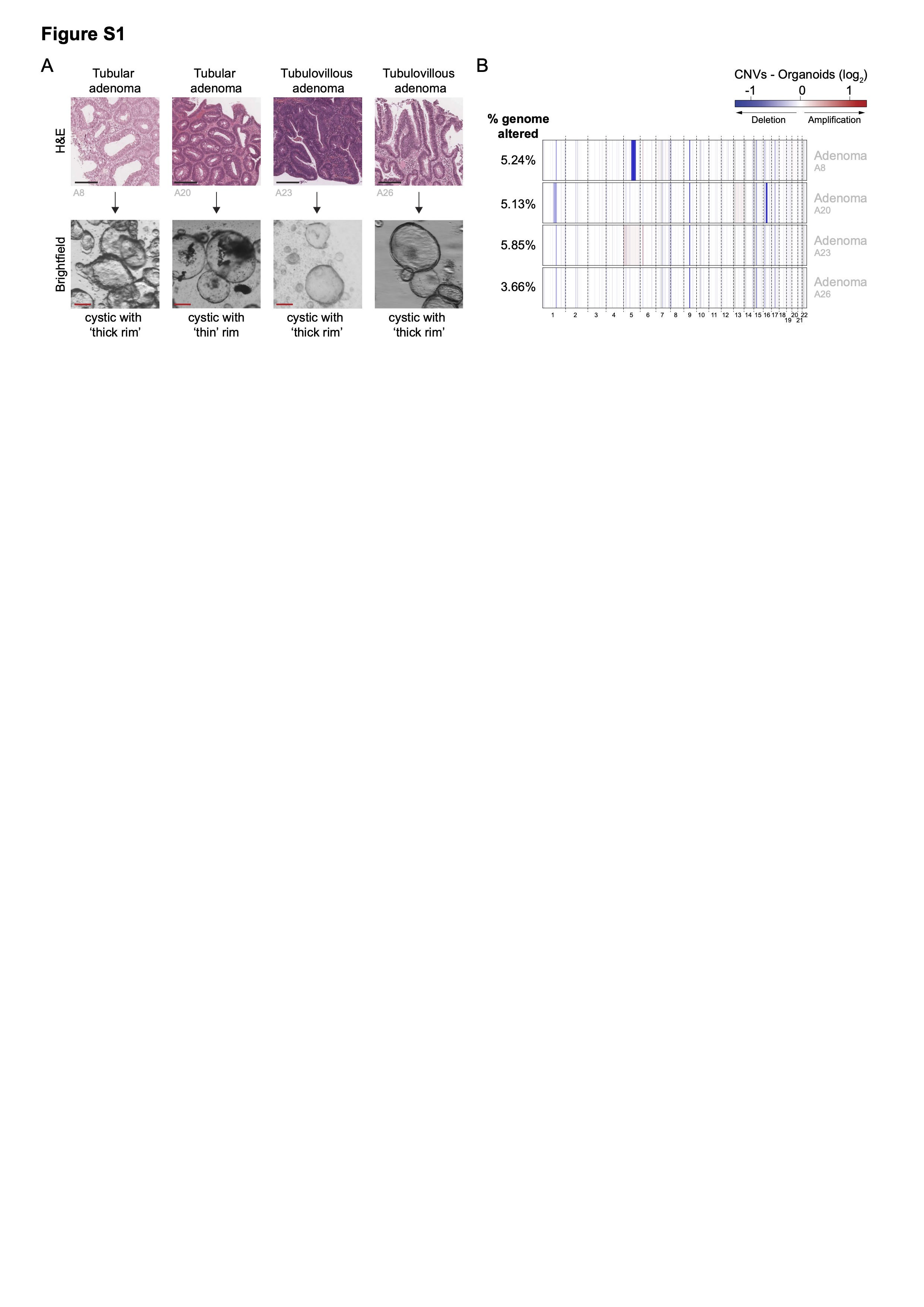

Supplement: Supplementary file 1 — Supplementary Material 1. [file 13046_2026_3666_MOESM1_ESM.zip › Figure S1.tiff]
